# A powerful Bayesian meta-analysis method to integrate multiple gene set enrichment studies

**DOI:** 10.1093/bioinformatics/btt068

**Published:** 2013-02-15

**Authors:** Min Chen, Miao Zang, Xinlei Wang, Guanghua Xiao

**Affiliations:** ^1^Quantitative Biomedical Research Center, Department of Clinical Sciences, The University of Texas Southwestern Medical Center, Dallas, TX 75390 and ^2^Department of Statistical Science, Southern Methodist University, Dallas, TX 75275, USA

## Abstract

**Motivation:** Much research effort has been devoted to the identification of enriched gene sets for microarray experiments. However, identified gene sets are often found to be inconsistent among independent studies. This is probably owing to the noisy data of microarray experiments coupled with small sample sizes of individual studies. Therefore, combining information from multiple studies is likely to improve the detection of truly enriched gene classes. As more and more data become available, it calls for statistical methods to integrate information from multiple studies, also known as meta-analysis, to improve the power of identifying enriched gene sets.

**Results:** We propose a Bayesian model that provides a coherent framework for joint modeling of both gene set information and gene expression data from multiple studies, to improve the detection of enriched gene sets by leveraging information from different sources available. One distinct feature of our method is that it directly models the gene expression data, instead of using summary statistics, when synthesizing studies. Besides, the proposed model is flexible and offers an appropriate treatment of between-study heterogeneities that frequently arise in the meta-analysis of microarray experiments. We show that under our Bayesian model, the full posterior conditionals all have known distributions, which greatly facilitates the MCMC computation. Simulation results show that the proposed method can improve the power of gene set enrichment meta-analysis, as opposed to existing methods developed by Shen and Tseng (2010, *Bioinformatics,* 26, 1316–1323), and it is not sensitive to mild or moderate deviations from the distributional assumption for gene expression data. We illustrate the proposed method through an application of combining eight lung cancer datasets for gene set enrichment analysis, which demonstrates the usefulness of the method.

**Availability:** http://qbrc.swmed.edu/software/

**Contact:** Min.Chen@UTSouthwestern.edu

**Supplementary information:** Supplementary data are available at *Bioinformatics* online.

## 1 INTRODUCTION

Although microarray analysis initially focused on identifying differentially expressed (DE) genes, increased attention has been paid to pathway or gene set analysis, which aims to detect altered biological pathways or other pre-defined gene classes rather than individual genes (e.g. Barry *et al.*, 2008; Efron and Tibshirani, 2007; Hosack *et al.*, 2003; Kim and Volsky, 2005; Subramaniana *et al.*, 2005; Tian *et al.*, 2005). Gene sets are usually defined based on gene functions, ontology information, chromosomal proximity or known regulatory relationships. Gene sets that are altered in responding to changes in phenotypes or treatments may provide important insights into molecular functions and gene regulatory relationships underlying biological processes. Also, it has been reported that gene set analysis tends to yield more reproducible and interpretable results than single gene analysis (Manoli *et al.,* 2006; Subramaniana *et al.,* 2005).

A major type of the gene set analysis is to determine whether a pre-defined category of genes is enriched (i.e. over-represented) by DE genes, often referred to as gene set (or pathway) enrichment analysis. A gene set is claimed to be enriched if it contains more DE genes than would be expected by chance. Many methods have been developed in gene set enrichment analysis for a single genomic study. For recent reviews, see Ackermann and Strimmer (2009), Hung *et al.* (2012) and the references therein.

**Fig. 1.**
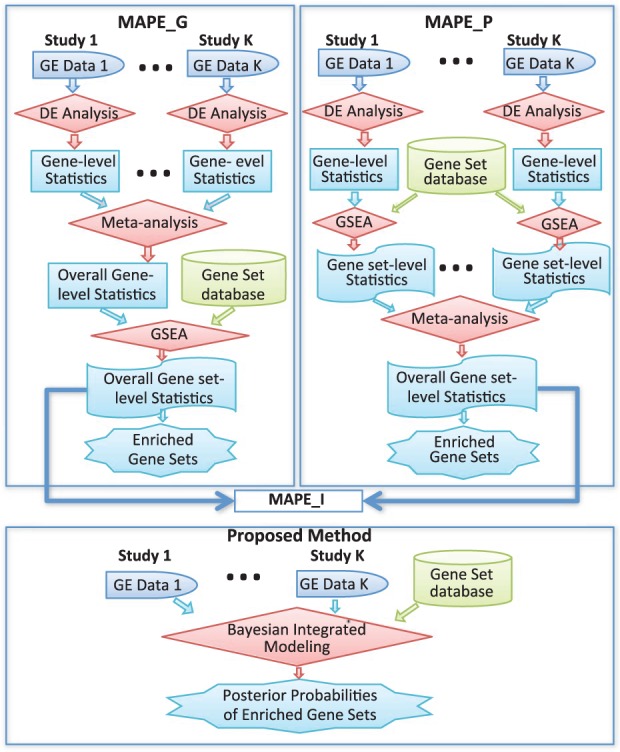
Flow charts of the MAPE algorithms and the proposed method. GE stands for gene expression; DE stands for differential expression and GSEA stands for Gene Set Enrichment Analysis (Subramaniana *et al.,* 2005). Note that MAPEs are all based on GSEA for enrichment analysis

Although plenty of gene expression data are publicly available now, it is challenging to integrate information of gene set enrichment analysis from multiple genomic studies targeting the same biological problem. Often sample sizes of individual studies are not large and microarray data are noisy, making estimation and inference highly variable. This often leads to inconsistent conclusions across studies. Integrative analysis of independent studies may facilitate information sharing and improve the power of detecting truly enriched gene classes, as well as increase reproducibility and interpretability. However, direct combination of multiple microarray datasets such as stacking them into one is extremely difficult owing to the incompatibility of data generated from various microarray platforms and different versions within the same platform (Mah *et al.,* 2004). Therefore, meta-analysis, a systematic statistical synthesis of data from multiple studies, has been widely used to aggregate evidence from related studies (Manoli *et al.,* 2006). Most meta-analyses currently target gene-level analysis with the exception of Shen and Tseng (2010) who, for the first time, systematically developed and evaluated meta-analysis for pathway enrichment analysis in microarray studies (Tseng *et al.*, 2012). They proposed three methods, namely, meta-analysis for pathway enrichment at gene level (MAPE_G), meta-analysis for pathway enrichment at pathway level (MAPE_P) and meta-analysis for pathway enrichment integrated (MAPE_I), all based on the popular method GSEA (Subramanian *et al.*, 2005) for enrichment analysis. All these methods consist of two key stages. The first is to conduct differential expression analysis to obtain gene-level statistics for each individual study. The second stage is to conduct pathway enrichment analysis and meta-analysis in either order, where the order distinguishes MAPE_G from MAPE_P: MAPE_G first conducts meta-analysis to combine the gene-level results over all the studies, and then conduct pathway enrichment analysis just once using the combined gene-level statistics; by contrast, MAPE_P conducts pathway enrichment analysis for each individual study and then combines the gene-set level results to get the overall gene-set statistics for a final conclusion. The third method MAPE_I further combines the end results from MAPE_G and MAPE_P for potential improvement in performance. See the top two panels of Figure 1 for the MAPE diagrams. Undoubtedly, by formalizing these simple and natural ideas, Shen and Tseng (2010) made an initial but important attempt in meta-analysis of gene set enrichment. However, such sequential approaches in analysis might cause information loss by just focusing on summary results from the previous steps without using all the expression data available. Further, when there exist various sources of heterogeneities among different datasets, these methods may lack power in detection without explicitly modeling the phenomena.

When data from component studies are available, using all data in a fully integrated model-based framework is in general more efficient than a sequential approach using summary statistics only. Specifically, the model should be able to account for between-study heterogeneities that are widely present in microarray data. Heterogeneities include, among others, varying experiment designs that lead to non-uniform inclusion of genes, unequal sample sizes and data qualities. Moreover, the gene expression measures in component studies may have different means and variances depending on platforms and pre-processing procedures. In this article, we propose a flexible Bayesian model that can offer an appropriate treatment of these problems when aggregating multiple studies to identify enriched gene sets. Our Bayesian method provides a natural way for data synthesis by incorporating model and parameter uncertainties involved in all studies. More importantly, it furnishes an integrated Bayesian framework for jointly modeling gene expression data from multiple studies and gene set information. This will allow researchers to conduct differential expression analysis, gene set enrichment analysis and meta-analysis simultaneously, all based on objective Bayesian posterior inference, which may yield more reliable scientific findings than the existing sequential approaches. See Figure 1 for comparison with the MAPE methods.

## 2 MODEL

Suppose there are *K* independent studies considered in a meta-analysis and *I_k_* samples in study *k*, where 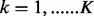. Let *J* be the total number of distinct genes in the genome of interest that appear in at least one of the *K* studies. Let *V_jk_* be an indicator variable for gene *j* being included in study *k*. Here, different studies are allowed to have different genes from the same genome, which offers great flexibility in the inclusion of potential studies for meta-analysis. Further, we assume that a gene can have different expression intensity measures but share the same DE/EE status in all constituent studies. In other words, a DE (EE) gene will always be differentially (equally) expressed in all the studies but the measurements of its expression can have different magnitudes across studies, which, again, permits more studies to be considered in the analysis.

Given 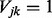, let *Y_ijk_* be the expression intensity (after pre-processing procedures and possibly some transformation) for gene *j* in sample *i* of study *k*, where 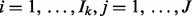 and 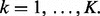 A binary phenotype label *X_ik_* is given to sample *i* of study *k*: 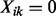 represents a control sample, and 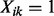 represents a case sample in study *k*. The mean expression intensity of a gene is assumed to be the same for all samples of each phenotype in a study, but in different studies the mean intensity can be different for the given gene. A common variance 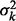 of measurement errors is assumed for all genes in study *k*. When the sample sizes are small or the signal-to-noise ratios are low in one or more studies, as is typical in many meta-analyses, this assumption would allow for information pooling across genes under a hierarchical Bayes setup, which is useful in stabilizing variance estimation. However, in other situations, the common variance assumption might be restrictive, and relaxing 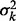
s to gene-specific 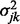
s can be done (see discussion in Section 6). The model is specified as follows:
(1)
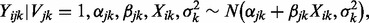

where 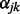 is the baseline expression level of gene *j* in study *k* (i.e. the mean intensity for the control samples), and 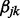 is the change in expression intensity between the different phenotypes. Let 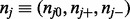 be a status indicator vector for gene *j*: if gene *j* is down-regulated (DR)/up-regulated (UR)/equally expressed (EE), then 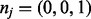/(0,1,0)/(1,0,0). Because the expression change 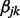 depends on gene *j*’s status, we assume that 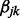 follow a normal mixture distribution with three modes, namely 0, 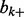 and 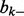, which correspond to EE, UR and DR genes, respectively: 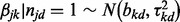, where 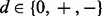, 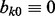 and 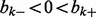. Further, we assume that the baseline 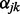 follow a normal distribution with study-specific mean and variance, namely 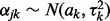. The indicator vector 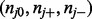 is assumed to follow a multinomial distribution with the parameter vector 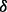: 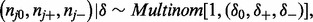 where 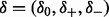 can be interpreted as the probability of EE, UR and DR genes in the overall gene list.

To account for uncertainties in the parameters introduced by the model and to avoid subjective inference, we specify non-informative priors on 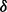, 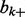, 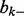 and *a_k_*: 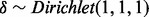, 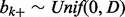, 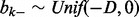, 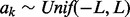, where *D* is chosen to be the maximum absolute value of changes in gene expression between different phenotypes in all *K* studies, and *L* is set to the maximum absolute value of gene expression with phenotype 0 in all *K* studies. Further, all variance components are given the Inverse-Gamma (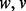) prior. Here*, w* and *v* are small numbers (e.g. 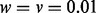) so that the prior density reflects vague prior knowledge on those variance parameters.

Now we proceed to model the gene set information. We denote the class of all pre-defined gene sets considered in the meta-analysis by 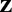, and let *G* be the total number of gene sets. We represent **Z** by a 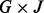 matrix in which all elements are binary variables. Let 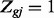, if gene *j* is in set *g* and 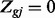 otherwise. Because we do not require 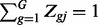 for each gene *j*, overlapping genes are allowed among different gene sets. Let 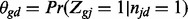 be the conditional probability that a gene is in set *g* given that the gene status is *d*, where 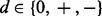. Then, for each set *g* and each gene *j*, the binary variable *Z_gj_* can be modeled by a Bernoulli distribution conditional on the status of gene *j*: 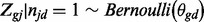, where 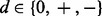. Here 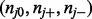 is the key vector that connects the gene sets with the expression data. If 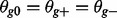 for set *g*, then *Z_gj_* is independent of 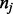; that is, whether any gene belongs to set *g* does not depend on whether the gene is EE/UR/DR. Further, if 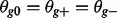 holds for all the gene sets, then the gene expression data **Y** (the collection of all *Y_ijk_*s) become independent of the gene set data **Z**. However, in many practical situations, genes within certain pre-defined gene sets (e.g. functional groups, biological pathways) are likely to be co-expressed, which leads to positive correlation in their expression levels (Pan, 2006; Wei and Pan, 2008; Wang *et al.,* 2012). These genes, if DE, tend to be altered in the same direction. Thus, 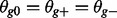 may not hold for some of the *G* gene sets so that **Y** and **Z** are linked together because both are conditional on 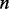 (the collection of all 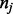
s). As will be shown later, through such a simple conditional probability setup, our model leads to an efficient Markov chain Monte Carlo (MCMC) algorithm and our proposed method compares favorably with competing methods.

Next, we assign Beta priors to 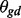
s for 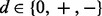 and 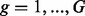: 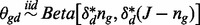, where *n_g_* is the number of genes in the gene set *g*, and 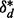 represents the true proportion of genes with status *d* in the overall gene list. Although the values of 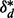
s are unknown, they can be roughly estimated from expression data using some existing method or specified directly using relevant biological knowledge, or they can be simply set to 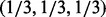 to reflect the non-informative prior belief. From the beta prior, we see that the prior mean of 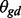 is set to be 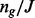, the proportion of genes in set *g* among the total gene list. That means the mean probability for a gene falling into set *g* is proportional to the size of the gene set *n_g_* and does not depend on the gene status. In this sense, the prior is sort of ‘non-informative’. The prior variance of 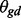 is 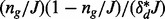. Thus, the larger 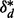 is (i.e. more genes with status *d*), the smaller the prior variance is. In practice, it is often believed that only a few genes are truly ‘interesting’, and most genes are EE. This prior specification would allow that 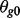 for EE genes is more concentrated around the prior mean 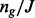 for set *g*, while 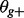 and 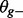 for DE genes have large prior variability around the mean. This seems to be reasonable.

## 3 POSTERIOR COMPUTATION AND BAYESIAN INFERENCE

Details about Bayesian computation, including the full probability model and full posterior conditionals, can be found in the Supplementary Material. Although not all the parameters are directly related to the detection of enriched gene sets, we need to use MCMC to draw random samples from the joint posterior distribution. Then statistical inference can be made by marginalizing over the posterior samples. One advantage of the proposed model is that the posterior conditionals for all the parameters involved, as listed above, are known distributions, from each of which direct sampling can be done. This property allows us to adopt an efficient Gibbs sampler, in which all the parameters are drawn sequentially and generated readily from the above conditional distributions without using any built-in sampling algorithm (such as Metropolis–Hastings and Acceptance/Rejection algorithms).

Here, our Bayesian inference is primarily focused on the gene set enrichment analysis. As mentioned in the introduction, an enriched gene set is defined as a set that has significantly higher percentage of DE genes than would be expected by chance. Or more formally, let 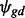 be the conditional probability that a gene has status *d* given that it is in set *g*. Then, a gene set *g* is claimed to be enriched if 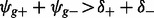.

Let 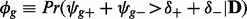 be the posterior probability that set *g* is enriched, where **D** represents all observed data. Let 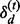 and 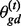 denote the posterior draws of the parameters 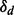 and 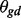 in the 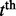 iteration of MCMC, for 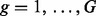 and 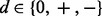. Then we can estimate 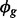 by 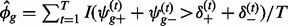, where *T* is the total number of iterations after the burn-in period; 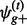 and 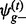 are calculated using the following relationship based on the Bayes rule: 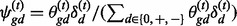. To identify enriched gene sets, we can rank all the gene sets based on 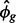 and select the sets on the top of the ranked list. The choice of a significance cutoff can be determined by controlling the Bayesian false discovery rate (*FDR*) (Newton *et al.,* 2004). For a given cutoff *c*, the *FDR* can be estimated by 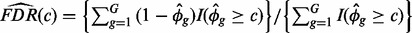, where 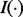 is the indicator function. We can choose *c* so that 
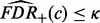
, where κ can be pre-specified (e.g. 1, 5, 10%).

Unlike the existing MAPE methods that are purely algorithm-based, our Bayesian model integrates all the information from different sources coherently, which enables us to conduct differential expression analysis, gene set enrichment analysis and meta-analysis in parallel rather than in a sequential manner. Further, it allows the use of objective decision rules based on posterior probabilities to detect enriched gene sets. The simple classification scheme makes the results from our Bayesian inference easy to interpret.

## 4 SIMULATION

We design three simulation studies to examine the performance of the proposed method in gene set enrichment meta-analysis, and compare it with the three existing methods, MAPE_G, MAPE_P and MAPE_I, developed by Shen and Tseng (2010). In the first study, we mainly follow the setup in Section 3.1 of Shen and Tseng (2010) that uses a single gene-set simulation model, and compare the power of the four methods in identifying the enriched gene set under different scenarios. In the second study, we extend the single gene-set model and consider multiple gene sets: some sets are enriched by UR genes and so are referred to as UR gene sets, some others are enriched by DR genes and so are referred to as DR gene sets, and all the remaining ones are non-enriched. This would allow us to compare the sensitivity and specificity of the four methods using receiver operating characteristic (ROC) curves. In the past study, we generate expression data from non-normal distributions to examine whether there is still an advantage of using the proposed method, as opposed to the three existing methods, when the normality assumption for gene expression intensities is violated.

In all our simulation, we follow Shen and Tseng (2010) and run MAPEs at their default setting. Also, when specifying the beta priors for the proposed method, we estimate 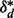
s from data using an overly simplistic approach so that the estimates are far from the true values sometimes. In fact, our experience from previous numerical studies suggests that the performance of our method is robust to the choice of 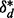
s. Thus, in practice, we suggest the non-informative setup 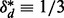 for simplicity.

### 4.1 Simulation I

To compare the power in gene set enrichment analysis using multiple studies, we apply the similar simulation settings as described in Shen and Tseng (2010). Suppose there are 500 genes in the genome and the first 100 genes are included in a gene set, which is the only one considered in this simulation. Shen and Tseng (2010) assumed that all DE genes involved are UR genes. To simulate more realistic situations, we assume there are 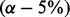 UR genes and 5% DR genes in the gene set. In the remaining 400 genes, there are 5% UR genes and 5% DR genes. Therefore, if 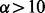
%, this gene set is enriched. Note that in Simulation I, we always include the case of 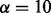
%, where the gene set is not enriched (i.e. the null case), to establish reference distributions of the statistics for the purpose of controlling the type I error. In each study there are 40 samples where the first 20 are control samples and the last 20 are case samples. Recall that we use a binary variable *V_jk_* to indicate whether gene *j* is included in study *k*. Here, we assume a universal sampling rate λ for different studies, where 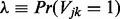, and we use Bernoulli(λ) to generate *V_jk_*s so that the number of genes in study *k* is random. All EE gene expression intensities are assumed to follow 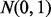, and we set 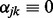 for all *k* and *j*. The UR genes in study *k* have expression intensities from 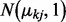 and DR genes are from 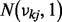, where 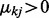 and 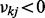. We set 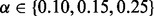 and 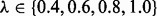.

As in Shen and Tseng (2010), five scenarios are considered in Simulation I. In the first three scenarios, we set both 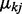 and 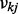 to be constant over *j* (i.e. genes) and to satisfy 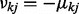 so that all the DE genes have a common absolute mean expression level, which intuitively represents the effect size (i.e. how strong the signal is). In the first scenario, two studies with the same effect size are considered; and we vary the degree of the effect size at two different levels. In the second scenario, again, two studies are considered but with different effect sizes. In the third, four studies instead of two are considered and everything else is the same as the first scenario. In the last two scenarios, we consider varying effect sizes across genes; that is, 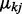 or 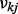 is no longer constant over *j* and they are generated from independent normal distributions with the same absolute mean. The fourth scenario considers two studies with the same mean of the effect sizes, while the fifth considers two studies with different means of the effect sizes. The details of the simulation settings are summarized in Supplementary Table S1.

In total, 100 independent datasets are generated for each fixed parameter set 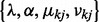. For each simulated dataset, we run MCMC and then calculate 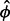 for our proposed method, and compute the Q values for the three MAPEs. For a fair comparison in power, we control the test size (i.e. the type I error) at the level 0.05 for all the methods. To do so, for each of the methods and each setting, we obtain the 5th percentile from the empirical distribution of the corresponding statistic [i.e. 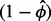 for the Bayesian model and Q values for the MAPEs] using the 100 datasets generated with the null-case parameter set 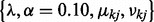, and use it as the cutoff value to declare whether the gene set is enriched or not. Then the power of each method is estimated by the proportion of datasets in which the gene set is found to be enriched.

**Fig. 2.**
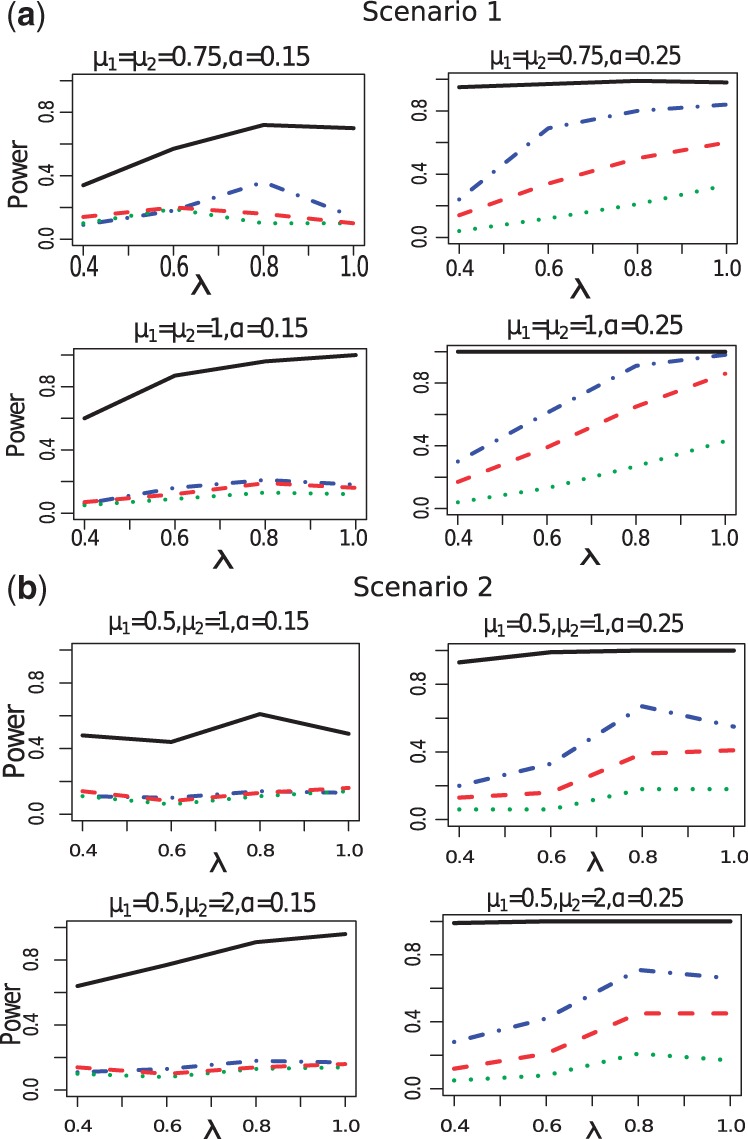
Power comparison for the first two scenarios in Simulation I. In each subpanel*,* the dash-dot line represents MAPE_P; the dotted line represents MAPE_G; the dash line represents MAPE_I and the solid line represents our Bayesian method

Figure 2 displays the power comparison results for Scenario 1–2. Figures of Scenario 3–5 are included in Supplementary Material (Supplementary Figs S1–S3). In all cases of Scenario 1, the proposed method has higher power than the MAPEs, especially when the enrichment signal is weak (i.e. the proportion α of DE genes in the gene set is relatively close to that in the whole genome). All methods tend to have increased power when λ increases. However, the power of the proposed method improves more rapidly than the MAPEs when α is 0.15, and it does not change much when α is 0.25 because the power is close to one regardless of λ. When the enrichment signal α is increased, all the four methods appear to have increased power. However, the MAPEs seem to be much more sensitive to the change of α than the Bayesian method. When the effect size is one, the power of the Bayesian method is almost one in nearly all the cases while the power of MAPE_P, the best of its kind, reaches one only when the sampling rate λ is one and the DE gene proportion of the gene set (α) is 0.25. We also test cases (results not reported) in which the effect size is more than one (2 or 4). We find that for large effect sizes, the Bayesian method always has power equal to one, while the MAPEs do not, although their performance improves substantially and the differences between the Bayesian method and MAPEs become small, especially for large λ or α. In real microarray studies, the signal-to-noise ratio may be low owing to the high noise level and may vary across different studies. Therefore, in Scenario 2, the effect size of one study is set to be relatively small. In this case we still have similar findings as Scenario 1. In Scenario 3 (Supplementary Fig. S1), as the number of studies increases from two to four, the power tends to increase for all the four methods compared with Scenario 1, and the Bayesian method still has higher power than the other three in nearly all the cases. Further, similar observations as in the first two scenarios can be made from results for Scenarios 4 and 5 (Supplementary Figs S2 and S3), where the effect sizes are random across genes in component studies.

### 4.2 Simulation II

We want to compare the sensitivity and specificity of our model with MAPEs when there are multiple gene sets including both non-enriched and enriched sets. Two studies are considered and each study contains 40 samples, of which the first 20 are controls and the remaining 20 are cases. Suppose there are 1000 genes in the genome. The first 200 genes are UR genes, the last 200 are DR genes and the middle 600 are EE genes. See Supplementary Table S2a for how we generate expression data for these genes. A total of 100 gene sets are generated in this simulation, where the first 30 sets are enriched by UR genes, the next 30 are enriched by DR genes and the last 40 are non-enriched sets. See Supplementary Table S2b for how we generate these gene sets. In each gene set, UR, DR and EE genes are randomly selected from their corresponding gene populations. We set 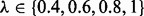 as before.

**Fig. 3.**
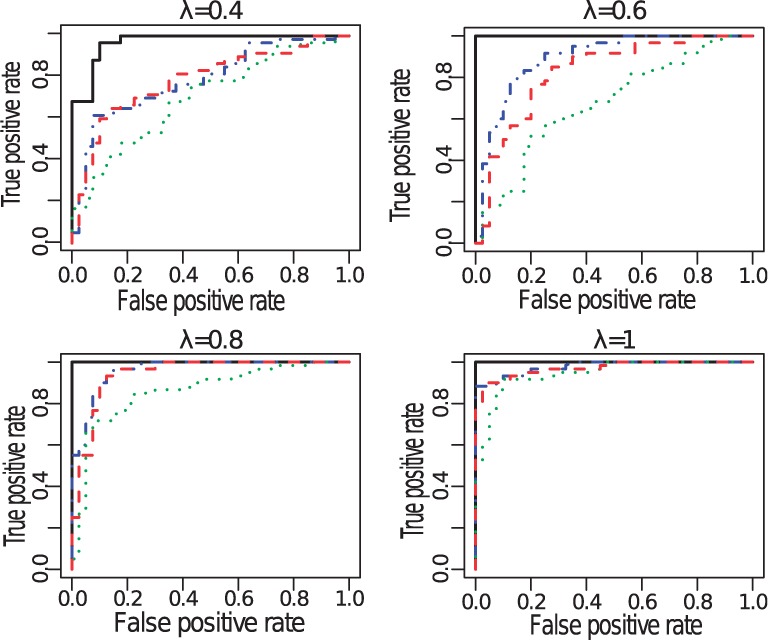
ROC curve comparison of Simulation II. In each subpanel*,* the dash-dot line represents MAPE_P; the dotted line represents MAPE_G; the dash line represents MAPE_I and the solid line represents our Bayesian method

The ROC curves for identifying enriched gene sets in Simulation II are shown in Figure 3. When the sampling rate λ is 1, all methods work well. However, when 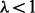, it is clear that the proposed method outperforms any of the MAPE methods. When the sampling rate (λ) is 0.4, the area under the curve (AUC) of the Bayesian method equals 0.97, while those of MAPE_P, MAPE_G and MAPE_I are 0.88, 0.67 and 0.85, respectively. If the sampling rate is 0.6 or 0.8, the ROC curve of the Bayesian model becomes perfect while the MAPEs do not. The results show that the proposed model is useful when the gene coverage is widely different among component studies in the meta-analysis, which agrees with the observation in Simulation I.

### 4.3 Simulation III

We examine the robustness of the proposed method to deviations from the normality assumption in (1) for gene expression intensities and compare its performance with MAPEs when this assumption is not satisfied. The simulation settings are the same as in Simulation II except that we simulate the expression values of DE genes for cases from t and Gamma distributions, instead of normal distributions. It is known that a t-distribution has heavier tails than the standard normal distribution and a Gamma distribution is skewed. For the t-distribution setting, the expression intensities for cases are simulated using a 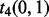 random variable plus a location shift. For the UR/DR genes, the shift is 2/−2 in study one and 1.5/−1.5 in study two. In the Gamma setting, expression intensities of UR genes for cases are from Gamma (2,2) in study one and from Gamma (1.5,2) for study two. The expression intensities of DR genes for cases are generated from the same Gamma distribution as UR genes in the same study, but with a negative sign. Similar to Simulation II, ROC curves (Supplementary Figs S4 and S5) are compared for all the four methods with the sampling rate 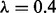, 0.6, 0.8 and 1, and their corresponding AUC values are displayed in Table 1. It is clear that the proposed Bayesian model uniformly outperforms the other three methods in both t and Gamma distribution settings for all λ except for 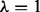, where all methods work well and have comparable performance. The simulation results suggest that the proposed Bayesian approach is not sensitive to mild or moderate deviations from the normality assumption for the expression intensities.

**Table 1.** AUC comparison in Simulation III

Distribution	λ	MAPE_P	MAPE_G	MAPE_I	Bayesian
t	0.4	0.88	0.72	0.85	1.00
	0.6	0.94	0.78	0.92	1.00
	0.8	0.96	0.92	0.96	1.00
	1	0.99	0.99	0.99	1.00
Gamma	0.4	0.79	0.70	0.79	1.00
	0.6	0.89	0.67	0.83	1.00
	0.8	0.96	0.86	0.94	1.00
	1	0.98	0.94	0.97	1.00

Expression intensities of DE genes for cases are drawn from t and Gamma distributions.

## 5 DATA EXAMPLE

The model is applied to eight independent lung cancer datasets and our goal is to find enriched gene sets/pathways related to lung cancer. All of the datasets are pre-processed by Robust Multi-array Average (RMA) (Irizarry *et al.,* 2003) and are log2-transformed. Patients in all datasets are classified into two groups based on their survival time, using the R package ‘pamr’ (Hastie *et al.,* 2011); see Supplementary Section S2 for detail. To avoid the heterogeneity in molecular mechanisms caused by the tumor subtypes, only patients with lung adenocarcinoma are considered. The names, sources and sample sizes of these datasets are displayed in Supplementary Table S3. A total of 186 C2 curated KEGG (Kanehisa *et al.,* 2012) pathways from MSigDB (Subramaniana *et al.,* 2005) are considered in this analysis. To better evaluate the results, 20 test gene sets, including 10 positive controls and 10 negative controls, are generated as follows. Positive control gene sets are from a list of genes that are believed to be highly related to the lung cancer (see Supplementary Table S4), while the negative control gene sets are randomly generated from the genes that are not included in the positive control gene list or involved in any KEGG gene set.

**Fig. 4.**
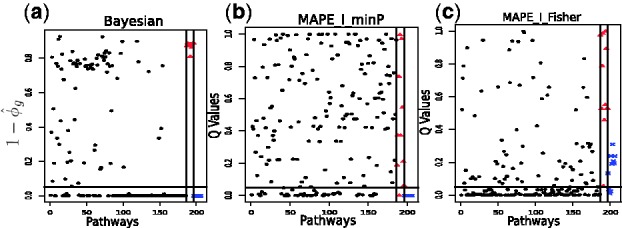
Scatter plot of (**a**) 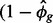) where 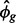 is the estimated posterior probability that pathway *g* is enriched; (**b**) Q-values from MAPE_I using Fisher’s statistic and (**c**) Q-values from MAPE_I using the minimum *P*-value statistic. The pathways represented by solid dots are the 186 KEGG pathways; those displayed as solid triangles are the 10 negative control gene sets and those represented by ‘x’ are the 10 positive control gene sets. The two vertical lines separate KEGG pathways*,* positive and negative controls. The horizontal line in (a) is the threshold corresponding to 
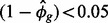
. The horizontal lines in (b) and (c) correspond to the threshold of Q-value <0.05

**Fig. 5.**
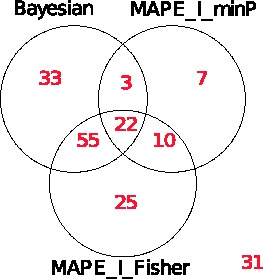
Venn diagram of enriched KEGG pathways identified by the three methods

We calculate 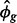
s, the estimated posterior enrichment probabilities defined in Section 3 from the MCMC samples, and plot 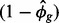 in Figure 4a. Convergence detection is done using standard graphic tools (trace and density plots) and the commonly used Gelman and Rubin diagnostic (Gelman and Rubin, 1992). For comparison, we first apply the three MAPE methods with the default option that uses the maximum *P*-value statistic, and find that none of them can identify any enriched pathways including those positive controls, even when we set the cutoff of the Q-values as high as 0.5. So we proceed to apply the MAPEs with two other available options: the minimum *P*-value statistic (minP) and the Fisher’s statistic (Fisher), and we find the MAPE_I method appears to perform better than or comparable with MAPE_P and MAPE_G in identifying positive controls while avoiding negative controls. So we report results for MAPE_I with the minimum *P*-value statistic (labeled MAPE_I_minP) and MAPE_I with the Fisher’s statistic (labeled MAPE_I_Fisher), and plot their Q-values in Figure 4b and c, respectively. We use 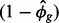 <0.05 in our Bayesian method, and use Q-value¡0.05 in the MAPE_I algorithms as the thresholds to find enriched pathways. Figure 4a clearly shows that, in the Bayesian method, the 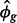
s for all positive control sets are equal to one while the 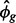
s for all negative control sets are close to or lower than 0.2. Thus, it correctly identifies all the positive controls as well as excluding all the negative controls. As shown in Figure 4b, MAPE_I_minP can identify all positive control pathways. However, it mistakenly claims that a negative control set is enriched. MAPE_I_Fisher fails to detect seven positive control sets although it correctly excludes all negative control sets (Fig. 4c). In Figure 5, we compare the numbers of enriched pathways via a Venn Diagram, which shows that the Bayesian model detects more enriched pathways than the MAPE_I methods.

A list of selected pathways identified by the proposed model are displayed in Supplementary Table S5 along with their posterior probabilities and Q-values of the two MAPE_I algorithms. These pathways can be classified into three groups. The first is the consensus group whose member pathways can be identified by all the three methods. Examples in this group include ‘non-small cell lung cancer’*, ‘*nucleotide excision repair’ and ‘DNA replication’, which play important roles in lung cancer. The second group of pathways are detectable by the Bayesian and MAPE_I_Fisher methods, but not by MAPE_I_minP. This group includes, for example*, ‘*pathways in cancer’ and ‘mTOR signaling pathway’. The ‘mTOR signaling pathway’ is an important signal transduction pathway in cell apoptosis and survival, and it has been a therapeutic target for lung cancer (LoPiccolo *et al.,* 2008). The third group contains those identified by the proposed method but missed by the two MAPE_I algorithms. For instance, ‘vascular endothelial growth factor (VEGF) signaling pathway’ belongs to this group. The VEGFs are known to play a prominent role during blood vessel formation. Importantly, tumor cells release VEGF that induces tumor neovascularization. Thus, this pathway is well established to be a target for antitumor therapy (Kowanetz and Ferrara, 2006). Another example is ‘transforming growth factor-beta (TGF-β) signaling pathway’. TGF-β and its signaling effectors are key factors in determining cancer cell behavior. It is reported that the TGF-β signaling pathway can act as a tumor suppressor as well as a promoter of tumor progression and invasion (Derynck *et al.,* 2001). While it took several previous studies to find these lung cancer-related pathways, our proposed method successfully captures them in one single meta-analysis. In addition, those pathways identified by the proposed method, but not yet discovered by existing studies, may be worth future biological investigation and validation.

**Fig. 6.**
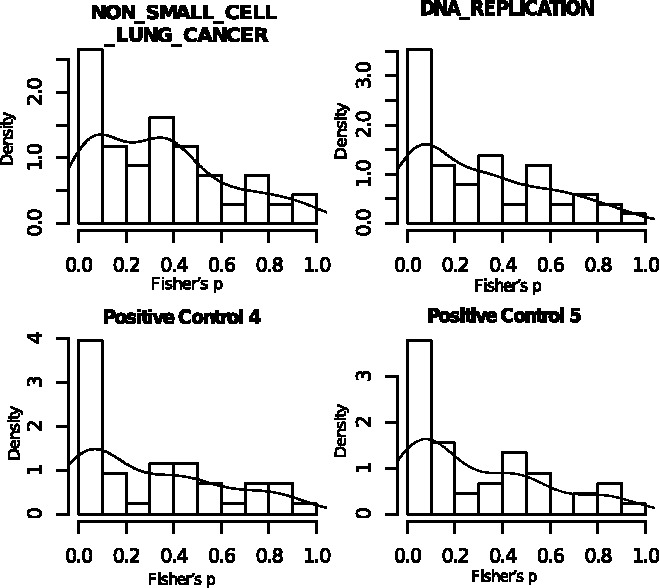
Empirical distributions of *P*-values from the Fisher’s combined probability tests for genes in selected pathways. The two pathways on the top have posterior enrichment probability estimated by one, and the bottom two are randomly chosen from the 10 positive control sets

To further examine the results from our Bayesian method, we use a popular method, SAM-t (Tusher *et al.,* 2001), to test differential messenger RNA expression and derive a *P*-value for each individual gene in each study. Then the Fisher’s combined probability test statistic, i.e. 
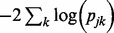
, is computed to combine the SAM-t *P*-values for each single gene from all studies. Finally, after performing a 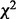 test, we obtain a *P*-value from the Fisher’s combined test for each gene. For genes in enriched sets, we would expect that many such *P*-values would be relatively low. Figure 6 shows *P*-values from the Fisher’s combined tests for genes in two pathways with estimated posterior probabilities of enrichment equal to one and two randomly chosen positive control sets. From Figure 6, we can see that the two identified pathways in the top panel and the two positive control sets in the bottom panel all have a large number of genes with small *P*-values. It indicates that these four pathways have high percentages of DE genes. Supplementary Figure S6 shows the Fisher’s *P*-values for the genes in another four pathways with estimated posterior enrichment probabilities equal to 0.09, 0.10, 0.13 and 0.11, respectively. Unlike the pathways in Figure 6, the distributions of *P*-values in these four pathways are not skewed to the left, suggesting that they are not likely to be enriched. This observation agrees with their low estimates of the posterior probabilities of enrichment.

## 6 DISCUSSION

We have proposed a fully integrated Bayesian model for meta-analysis of gene set enrichment using multiple genomic studies, and developed an efficient Gibbs sampler for posterior computation and inference, where all the steps can be done by direct sampling from known distributions. Through simulation studies and experimental data, we have shown that compared with the existing methods, our approach can substantially improve the power of detecting enriched gene sets, especially for non-easy situations when the effect size is not large, gene overlapping rate is low or enrichment signal is weak. The performance gain of the proposed method may be attributed to the following ideas behind our model-based approach: (i) explicit modeling of between-study heterogeneities for gene expression data; (ii) the capability of including non-overlapping genes in the model; and (iii) joint modeling of gene set information and expression data from multiple studies, which may utilize the available information more efficiently than the MAPEs that synthesize summary statistics only. Note that (iii) is closely related to our previous work (Wang *et al.,* 2012) but the focus of that paper was on a single genomic study with different data structures. Another advantage of our method is that it does not need the so-called gene-sampling scheme. As argued in Goeman and Buhlmann (2007), this scheme is implicitly required for computation or correct interpretation of *P*-values in the GSEA-like methods or 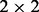 table methods, and is subject to the criticism that it is unrealistic. Instead, our Bayesian model makes every assumption clear; and it relies on posterior probabilities for identifying enriched gene sets, which reflect uncertainties after observing data and can be interpreted naturally without the gene-sampling scheme.

Computational efficiency is an important aspect of Bayesian modeling. Here we report the computing time of our simulation studies. It takes between 3 and 10 min, depending on the sampling rate λ, to run 4000 MCMC iterations for each dataset involving two studies in Simulation I using one thread on a Red Hat Enterprise Linux workstation with 4 Xeon(R) CPUs @2.67 GHz and 5.8 GB of memory. We also monitor the convergence of the MCMC processes, and the results suggest that the proposed Bayesian model converges relatively fast. In addition, under the non-informative prior setups, our algorithm can become a fully automated procedure that does not require any tuning parameter. This is convenient for an end user with little or no statistical training. However, if meaningful prior knowledge is available, certain features of the proposed method may be changed to produce potentially better results that incorporate such knowledge.

In a nutshell of the Bayesian framework, our method can be extended and some of the model assumptions can be relaxed, to allow for more modeling flexibility. First, motivated by the application in Section 5, we assume a common variance of error for all genes in study *k*. This is to avoid over-parameterization and help the variance estimation when some studies in meta-analysis lack enough samples to produce stable estimates of the gene-wise variances. However, for individual studies with sufficient samples, this assumption can be relaxed by using gene-specific variances 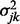
s instead of 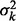 in (1). The Gibbs sampler can be modified readily; see the end of Supplementary Section S1 for detailed changes. Second, with minor adaption in the model and algorithm, the method can be also applied to paired expression data (such as those generated using two-channel arrays) besides two-sample expression data discussed in the article; see Chapter 4 of Zang (2012) for detail. Last, we assume that a gene shares the same differential status across studies as in the MAPE methods. This assumption may be plausible when all the studies in meta-analysis address the exactly same underlying biological question and they are conducted in similar experimental conditions. On the other hand, there exist situations where the true status of some genes can vary across studies. If one applies the proposed method directly here, these genes may need to be removed, when possible, from the gene pool before application, as suggested by one of the reviewers. To formally handle such situations, we can extend our Bayesian model by adding a layer of hierarchy (e.g. model the study-specific status of gene *j* as a random variable with probability 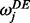 to be DE in each study). Such extension would change the model, the joint posterior distribution and MCMC algorithm. Clearly, there is ample space for future work to deal with this issue.

In meta-analysis, it is critical to carefully review and choose studies because inclusion of data with poor quality might lead to biased results or loss of efficiency. Thus, we recommend readers to follow strict procedures of critical review of the literature before applying any meta-analysis model. Finally, we mention that one limitation of the proposed method is that, unlike MAPEs, binary phenotypes/conditions are required in our analysis. Nonetheless, there exist a wide range of applications that meet this requirement.

*Funding:* This work was supported by grants from National Institutes of Health (1K25AR063761, 4R33DA027592); National Science Foundation (DMS-0906545, DMS-0907562); National Aeronautics and Space Administration (NNJ05HD36G, NNX09AM08G); and Cancer Prevention Research Institute of Texas (RP101251, RP120840).

*Conflict of Interest*: none declared.

## Supplementary Material

Supplementary Data

## References

[btt068-B1] Ackermann M, Strimmer K (2009). A general modular framework for gene set enrichment analysis. BMC Bioinformatics.

[btt068-B2] Barry WT (2008). A statistical framework for testing functional categories in microarray data. Ann. Appl. Stat..

[btt068-B3] Derynck R (2001). TGF-beta signaling in tumor suppression and cancer progression. Nat. Genet..

[btt068-B4] Efron B, Tibshirani R (2007). On testing the significance of sets of genes. Ann. Appl. Stat..

[btt068-B5] Gelman A, Rubin DB (1992). Inference from iterative simulation using multiple sequences. Stat. Sci..

[btt068-B6] Goeman JJ, Buhlmann P (2007). Analyzing gene expression data in terms of gene sets: methodological issues. Bioinformatics.

[btt068-B7] Hastie T (2011). http://cran.r-project.org/web/packages/pamr/index.html.

[btt068-B8] Hosack DA (2003). Identifying biological themes within lists of genes with ease. Genome Biol..

[btt068-B9] Hung JH (2012). Gene set enrichment analysis: performance evaluation and usage guidelines. Brief. Bioinform..

[btt068-B10] Irizarry RA (2003). Summaries of affymetrix genechip probe level data. Nucl. Acids Res..

[btt068-B11] Kanehisa M (2012). KEGG for integration and interpretation of large-scale molecular data sets. Nucleic Acids Res..

[btt068-B12] Kim SY, Volsky DJ (2005). Page: parametric analysis of gene set enrichment. BMC Bioinformatics.

[btt068-B13] Kowanetz M, Ferrara N (2006). Vascular endothelial growth factor signaling pathways: therapeutic perspective. Clin. Cancer Res..

[btt068-B14] LoPiccolo J (2008). Targeting the PI3K/Akt/mTOR pathway: effective combinations and clinical considerations. Drug Resist. Updat..

[btt068-B15] Mah N (2004). A comparison of oligonucleotide and cDNA-based microarray systems. Genomics.

[btt068-B16] Manoli T (2006). Group testing for pathway analysis improves comparability of different microarray datasets. Bioinformatics.

[btt068-B17] Newton MA (2004). Detecting differential gene expression with a semiparametric hierarchical mixture method. Biostatistics.

[btt068-B18] Pan W (2006). Incorporating gene functional annotations in detecting differential gene expression. J. R. Stat. Soc. Ser. C.

[btt068-B19] Shen K, Tseng GC (2010). Meta-analysis for pathway enrichment analysis when combining multiple genomic studies. Bioinformatics.

[btt068-B20] Subramanian A (2005). Gene set enrichment analysis: a knowledge-based approach for interpreting genome-wide expression profiles. Proc. Natl Acad. Sci. USA.

[btt068-B21] Tian L (2005). Discovering statistically significant pathways in expression profiling studies. Proc. Natl Acad. Sci. USA.

[btt068-B22] Tseng GC (2012). Comprehensive literature review and statistical considerations for microarray meta-analysis. Nucleic Acids Res..

[btt068-B23] Tusher VG (2001). Significance analysis of microarrays applied to the ionizing radiation response. Proc. Natl Acad. Sci. USA.

[btt068-B24] Wang X (2012). Bayesian joint analysis of gene expression data and gene functional annotations. Stat. Biosci..

[btt068-B25] Wei P, Pan W (2008). Incorporating gene functions into regression analysis of dna-protein binding data and gene expression data to construct transcriptional networks. *IEEE/ACM Trans. Comput*. Biol. Bioinform..

[btt068-B26] Zang M (2012). Bayesian Meta-analysis in Pathway Enrichment Analysis. *PhD Thesis*.

