# Supplementary material for: A powerful Bayesian meta-analysis method to integrate multiple gene set enrichment studies
Source: Bioinformatics. 2013 Feb 15;29(7):862–9. doi: 10.1093/bioinformatics/btt068 (PMC3605602; doi:10.1093/bioinformatics/btt068)
Supplement: Supplementary Data [file supp_29_7_862__index.html]

A powerful Bayesian meta-analysis method to integrate multiple gene set enrichment studies — A powerful Bayesian meta-analysis method to integrate multiple gene set enrichment studies — Supplementary Data 

# A powerful Bayesian meta-analysis method to integrate multiple gene set enrichment studies

## Supplementary Data

files

**Files in this Data Supplement:**

- Supplementary Data - pdf file
