# Supplementary material for: A powerful Bayesian meta-analysis method to integrate multiple gene set enrichment studies
Source: Bioinformatics. 2013 Feb 15;29(7):862–9. doi: 10.1093/bioinformatics/btt068 (PMC3605602; doi:10.1093/bioinformatics/btt068)
Supplement: Supplementary Data [file supp_btt068_Meta_GSEA-suppl_v4.pdf]

Supplementary Material

A powerful Bayesian meta-analysis method to integrate multiple gene set enrichment studies

S1 Details of Posterior Computation

Let $\boldsymbol{\tau}_k^2 = \left(\tau_{k0}^2, \tau_{k+}^2, \tau_{k-}^2, \tau_k^2\right)$ and $\boldsymbol{\theta}_g = (\theta_{g0}, \theta_{g+}, \theta_{g-})$. Let $J_k = \sum_{j=1}^J V_{jk}$ be the number of genes in study k, $N_d = \sum_{j=1}^J n_{jd}$ be the number of genes with status d in the total gene list, and $N_{kd} = \sum_{j=1}^J n_{jd} V_{jk}$ be the number of genes with status d in study k, where $k = 1, ...K$ and $d \in \{0, +, -\}$. The collection of all X_{ik}s is denoted by $\mathbf{X}$, the collection of all V_{jk}s is denoted by $\mathbf{V}$; and the collection of all parameters involved in the model is denoted by Θ. Then given $\mathbf{X}$ and $\mathbf{V}$, the full probability model is given by

$$
\begin{aligned}
p\left(\mathbf{Y}, \mathbf{Z}, \Theta | \mathbf{X}, \mathbf{V}\right) \propto & \prod_{k=1}^{K} \left\{ \prod_{j=1}^{J} \left\{ \left[\prod_{i=1}^{I_k} p\left(Y_{ijk} | X_{ik}, \beta_{jk}, \alpha_{jk}, \sigma_k^2\right) \right] p\left(\beta_{jk}, \alpha_{jk} | \boldsymbol{n}_j, \boldsymbol{\tau}_k^2, b_{k+}, b_{k-}, a_k\right) \right\}^{V_{jk}} \right\} \\
& \times \prod_{j=1}^{J} \left\{ \left[\prod_{g=1}^{G} p\left(Z_{gj} | \boldsymbol{n}_j, \boldsymbol{\theta}_g\right) \right] p\left(\boldsymbol{n}_j | \boldsymbol{\delta}\right) p\left(\boldsymbol{\theta}_g\right) \right\} p\left(\boldsymbol{\delta}\right) \times \prod_{k=1}^{K} \left[p\left(\boldsymbol{\tau}_k^2\right) p\left(b_{k+}\right) p\left(b_{k-}\right) p\left(\sigma_k^2\right) p\left(a_k\right) \right] \\
\propto & \prod_{k=1}^{K} \left\{ \left[\frac{1}{\sigma_k^{I J_k}} \exp\left[- \frac{\sum_i \sum_j \left(Y_{ijk} - \beta_{jk} X_{ik} - \alpha_{jk}\right)^2 V_{jk}}{2\sigma_k^2} \right] \right] \right\} \\
& \times \prod_{k=1}^{K} \left\{ \frac{1}{\tau_{k0}^{N_{k0}} \tau_{k+}^{N_{k+}} \tau_{k-}^{N_{k-}} \tau_k^{J_k}} \prod_{j=1}^{J} \exp\left[- \sum_{d \in \{0,+,-)\}} \frac{V_{jk} n_{jd} \left(\beta_{jk} - b_{kd}\right)^2}{2\tau_{kd}^2} - \frac{V_{jk} \left(\alpha_{jk} - a_k\right)^2}{2\tau_k^2} \right] \right\} \\
& \times \prod_{g=1}^{G} \prod_{d \in \{0,+,-\}} \left\{ \theta_{gd}^{\sum_j Z_{gj} n_{jd} + \delta_d^* n_g - 1} \left(1 - \theta_{gd}\right)^{\sum_j (1 - Z_{gj}) n_{jd} + \delta_d^* (J - n_g) - 1} \right\} \delta_0^{N_0} \delta_+^{N_+} \delta_-^{N_-} \\
& \times \prod_{k=1}^{K} \left\{ \left(\sigma_k^2 \tau_k^2\right)^{-w-1} \exp\left(- \frac{v}{\sigma_k^2} - \frac{v}{\tau_k^2} \right) \prod_{d \in \{0,+,-\}} \left(\tau_{kd}^2\right)^{-w-1} \exp\left(- \frac{v}{\tau_{kd}^2} \right) \right\}. \qquad \text{(S1)}
\end{aligned}
$$

To simplify the notations, we use $\Theta_{/\theta}$ to denote the parameter set that includes all the parameters except for θ. The following list gives the full posterior conditionals derived from the full probability model ($i = 1, ..., I_k$, $j = 1, ..., J$, $k = 1, ..., K$, $d \in \{0, +, -\}$); and R code for the corresponding Gibbs sampler is provided at the URL http://qbrc.swmed.edu/software/a-powerful-bayesian-meta-analysis-method-to-integrate-multiple-gene-set-enrichment-studies/

$$\beta_{jk} \mid V_{jk}=1, \Theta_{/\beta_{jk}} \sim N\left(\frac{\frac{\sum_{i=1}^{I_k}\left(Y_{ijk}-\alpha_{jk}\right)X_{ik}}{\sigma_k^2}+\sum_{d\in\{0,+,-\}}\frac{n_{jd}b_{kd}}{\tau_{kd}^2}}{\frac{\sum_{i=1}^{I_k}X_{ik}}{\sigma_k^2}+\sum_{d\in\{0,+,-\}}\frac{n_{jd}}{\tau_{kd}^2}}, \frac{1}{\frac{\sum_{i=1}^{I_k}X_{ik}}{\sigma_k^2}+\sum_{d\in\{0,+,-\}}\frac{n_{jd}}{\tau_{kd}^2}}\right), \tag{S2}$$

$$\alpha_{jk} \mid V_{jk}=1, \Theta_{/\alpha_{jk}} \sim N\left(\frac{\frac{\sum_i\left(Y_{ijk}-\beta_{jk}X_{ik}\right)}{\sigma_k^2}+\frac{a_k}{\tau_k^2}}{\frac{I_k}{\sigma_k^2}+\frac{1}{\tau_k^2}}, \frac{1}{\frac{I_k}{\sigma_k^2}+\frac{1}{\tau_k^2}}\right), \tag{S3}$$

$$\tau_{kd}^2 \mid \Theta_{/\tau_{kd}^2} \sim IG\left(\frac{N_{kd}}{2}+w, \frac{\sum_j n_{jd}V_{jk}\left(\beta_{jk}-b_{kd}\right)^2}{2}+v\right),$$

$$\tau_k^2 \mid \Theta_{/\tau_k^2} \sim IG\left(\frac{J_k}{2}+w, \frac{\sum_j V_{jk}\left(\alpha_{jk}-a_k\right)^2}{2}+v\right),$$

$$b_{k+} \mid \Theta_{/b_{k+}} \sim Truncated\,Normal\left(\frac{\sum_{j=1}^{J}V_{jk}n_{j+}\beta_{jk}}{N_{k+}}, \frac{\tau_{k+}^2}{N_{k+}}\right), \quad 0<b_{k+}<D,$$

$$b_{k-} \mid \Theta_{/b_{k-}} \sim Truncated\,Normal\left(\frac{\sum_{j=1}^{J}V_{jk}n_{j-}\beta_{jk}}{N_{k-}}, \frac{\tau_{k-}^2}{N_{k-}}\right), \quad -D<b_{k-}<0,$$

$$a_k \mid \Theta_{/a_k} \sim Truncated\,Normal\left(\frac{\sum_{j=1}^{J}V_{jk}\alpha_{jk}}{J_k}, \frac{\tau_k^2}{J_k}\right), \quad -L<a_k<L,$$

$$\sigma_k^2 \mid \Theta_{/\sigma_k^2} \sim IG\left(\frac{I_kJ_k}{2}+w, \frac{\sum_{i=1}^{I_k}\sum_{j=1}^{J}V_{jk}\left(Y_{ijk}-\beta_{jk}x_{ik}-\alpha_{jk}\right)^2}{2}+v\right), \tag{S4}$$

$$\theta_{gd} \mid \Theta_{/\theta_{gd}} \sim Beta\left(\sum_{j=1}^{J}Z_{gj}n_{jd}+\delta_d^*n_g, \sum_{j=1}^{J}\left(1-Z_{gj}\right)n_{jd}+\delta_d^*(J-n_g)\right),$$

$$\boldsymbol{\delta} \sim Dirichlet\left(N_0 + 1,\ N_+ + 1,\ N_- + 1\right),$$

$$\boldsymbol{n}_j \sim Multinomial\left(1,\ \frac{q_{j0}}{q_{j0} + q_{j+} + q_{j-}},\ \frac{q_{j+}}{q_{j0} + q_{j+} + q_{j-}},\ \frac{q_{j-}}{q_{j0} + q_{j+} + q_{j-}}\right),$$

with

$$q_{jd} = \delta_d \cdot \prod_{k=1}^{K} \left\{ \frac{1}{\tau_{kd}} \exp\left[-\frac{\left(\beta_{jk} - b_{kd}\right)^2}{2\tau_{kd}^2} \right] \right\}^{V_{jk}} \cdot \prod_{g=1}^{G} \left\{ \theta_{gd}^{Z_{gj}} \left(1 - \theta_{gd}\right)^{1 - Z_{gj}} \right\}.$$

Next, we relax the assumption that a common variance of error σ_k^2 is shared by all genes in study k, and outline the changes in the full conditionals. That is, we replace σ_k^2 by σ_{jk}^2s when study k has sufficient samples to produce stable estimates of the gene-wise variances. Then for such studies (say k),

1. When sampling β_{jk}s and α_{jk}s given $V_{jk} = 1$, replace σ_k^2 by σ_{jk}^2 in (S2) and (S3).
2. The step of sampling σ_k^2 should be replaced by the step of sampling σ_{jk}^2s. That is, (S4) should be replaced by the following:

$$\sigma_{jk}^2 \mid V_{jk} = 1, \Theta_{/\sigma_{jk}^2} \sim IG\left(\frac{I_k}{2} + w,\ \frac{\sum_{i=1}^{I_k} \left(Y_{ijk} - \beta_{jk} x_{ik} - \alpha_{jk}\right)^2}{2} + v \right),$$

   for any gene j with $V_{jk} = 1$, under independent Inverse-Gamma(w, v) priors for all σ_{jk}^2s.

3. All the other steps remain the same as before.

S2 Additional Tables and Figures from Simulation

Gene Type	Study	Para.	Scenarios of Simulation I				
			1	2	3	4	5
UR genes	1	μ_{1j}	$\{0.75, 1\}$	0.5	$\{0.75, 1\}$	$N(u, 0.5)$, $u \in \{1, 1.5\}$	$N(1, 0.5)$
	2	μ_{2j}	μ_{1j}	$\{1, 1.5\}$	μ_{1j}	$N(u, 0.5)$	$N(u, 0.5)$, $u \in \{1.5, 2\}$
	3	μ_{3j}	-	-	μ_{1j}	-	-
	4	μ_{4j}	-	-	μ_{1j}	-	-
DR genes	1	ν_{1j}	$-\mu_{1j}$	$-\mu_{1j}$	$-\mu_{1j}$	$N(v, 0.5)$, $v = -u$	$N(-1, 0.5)$
	2	ν_{2j}	$-\mu_{2j}$	$-\mu_{2j}$	$-\mu_{2j}$	$N(v, 0.5)$, $v = -u$	$N(v, 0.5)$, $v = -u$
	3	ν_{3j}	-	-	$-\mu_{3j}$	-	-
	4	ν_{4j}	-	-	$-\mu_{4j}$	-	-
	All	α	$\{0.10, 0.15, 0.25\}$				
	All	λ	$\{0.4, 0.6, 0.8, 1.0\}$				

Table S1: Simulation I settings of five scenarios. In the first scenario, two studies with the same effect size are considered; in the second, two studies are considered but with different effect sizes. In the third, four studies instead of two are considered and everything else is the same as the first scenario. The last two scenarios consider varying effect sizes across genes, where the fourth considers two studies with the same mean of the effect sizes while the fifth considers two studies with different means of the effect sizes.

(a) Gene expression for cases

Gene ID	Study 1	Study 2
1-200	$N(1, 1)$	$N(1.5, 1)$
201-800	$N(0, 1)$	$N(0, 1)$
801-1000	$N(-1, 1)$	$N(-1.5, 1)$

(b) Gene sets

Set ID	UR genes	DR genes	EE genes
1-30	35%	15%	50%
31-60	15%	35%	50%
61-100	20%	20%	60%

Table S2: Simulation II settings: (a) Genes 1-200 are UR genes, 201-800 are EE genes, and 801-1000 are DR genes; (b) Gene sets 1-30 are UR gene enriched sets, 31-60 are DR gene enriched sets, and 61-100 are non-enriched gene sets. Note that gene expression intensities for cases are simulated using the table in (a) while the intensities for controls are all simulated from $N(0, 1)$.

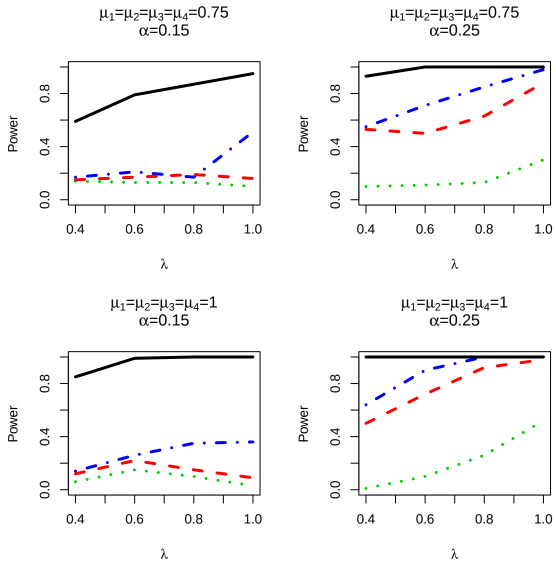

Figure S1: Simulation I–Power comparison for Scenario 3. In each subpanel, the blue dash-dot line represents MAPE_P; the green dotted line represents MAPE_G; the red dash line represents MAPE_I; and the black solid line represents our Bayesian method.

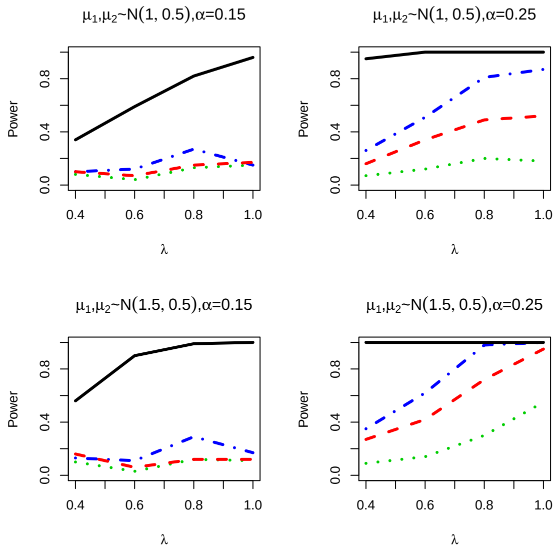

Figure S2: Simulation I–Power comparison for Scenario 4. In each subpanel, the blue dash-dot line represents MAPE_P; the green dotted line represents MAPE_G; the red dash line represents MAPE_I; and the black solid line represents our Bayesian method.

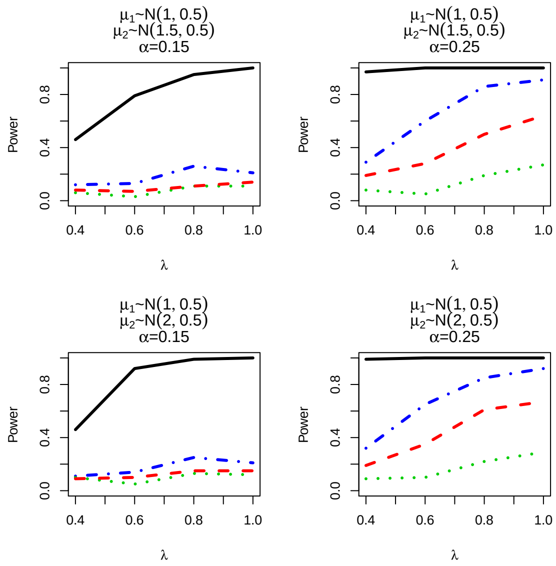

Figure S3: Simulation I–Power comparison for Scenario 5. In each subpanel, the blue dash-dot line represents MAPE_P; the green dotted line represents MAPE_G; the red dash line represents MAPE_I; and the black solid line represents our Bayesian method.

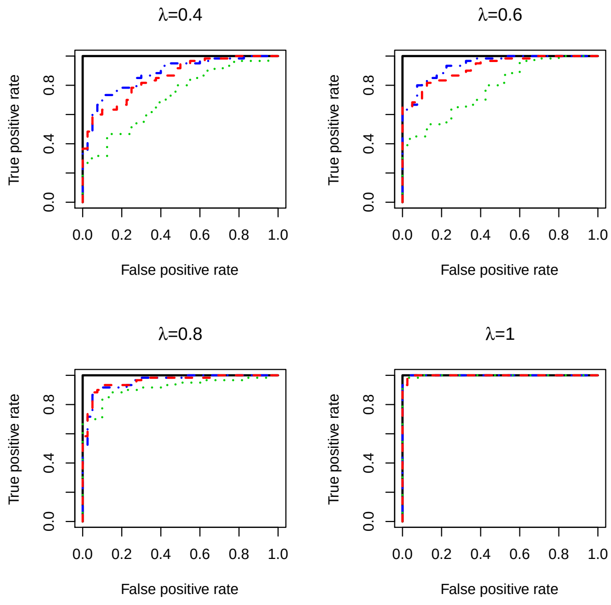

Figure S4: Simulation III–ROC curve comparison with the expression intensities of DE genes for cases from t-distributions with $df = 4$ (with heavier tails than normal distributions). In each subpanel, the blue dash-dot line represents MAPE_P; the green dotted line represents MAPE_G; the red dash line represents MAPE_I; and the black solid line represents our Bayesian method.

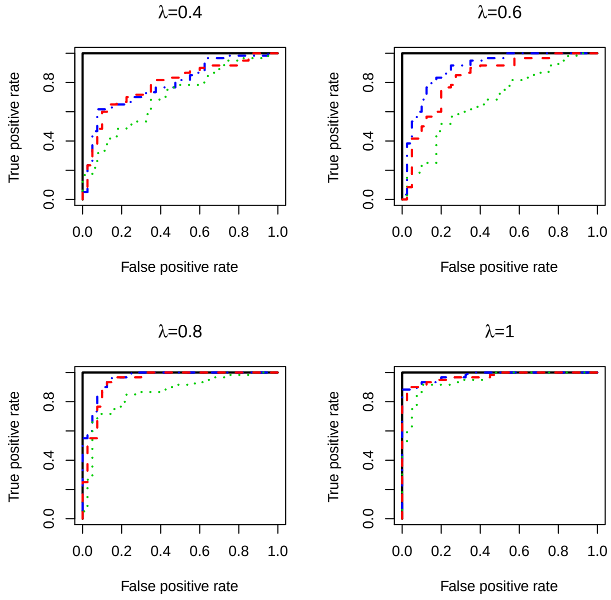

Figure S5: Simulation III–ROC curve comparison with the expression intensities of DE genes for cases from gamma distributions (skewed compared to normal distributions). In each subpanel, the blue dash-dot line represents MAPE_P; the green dotted line represents MAPE_G; the red dash line represents MAPE_I; and the black solid line represents our Bayesian method.

S3 Additional Tables and Figures from Data Analysis

In our data example, patients in all data sets were classified into two groups based on their survival time. We used the *pamr.surv.to.class2* function in the pamr R package (Tibshirani et al. 2002; Tibshirani et al. 2003) to determine the two groups (i.e., long and short survival groups). The function splits observations into the two groups based on the Kaplan-Meier estimates. For each observation (survival time, censoring status), it computes the probability of that observation falling into one of the two survival groups. The probability is 1 or 0 for an uncensored observation depending on the survival time. For a censored observation, the probability is between 0 and 1 based on the Kaplan Meier estimate.

The following are tables and figures for the data example.

Data Set Name	Number of (Controls, Cases)
GSE10245 (Kuner et al. 2009)	13, 27
GSE14814 (Zhu et al. 2010)	7, 21
GSE3141 (Bild et al. 2006)	22, 36
GSE3593 (Potti et al. 2006)	12, 31
CL (Shedden et al. 2008)	17, 65
Moff (Shedden et al. 2008)	27, 52
NCI_U133A (Shedden et al. 2008)	18, 86
NCI_Lung_U133A (Shedden et al. 2008)	44, 131

Table S3: Data example–Lung cancer data sets used in real data analysis. Data set GSE10245 and GSE 3141 have 20633 genes and the other six have 12992 genes.

DOCK9	SNRPA1	SLC35A5	FCF1
RRM2	DDX17	H3F3A	ABCC10
AURKA	MRPL3	HNRNPK	SFTPB
HOPX	HMGB2	HSPD1	BTF3
PRC1	CYCS	DEK	CYB5A
GPR116	NFIB	CBX3	YPEL5
NKX2-1	ATP5I	NBPF15	UBE2J1
TTC37	RGL1	ATP1B1	HIGD1A
CDKN3	CBLB	NDUFAB1	NUSAP1
COL4A3	ZMYM2	NUP153	CCDC90B
IFT57	TUBA1B	CTSH	CCDC59
ATP8A1	BLVRA	KIAA0101	OLA1
C1orf116	LARP1	PLOD2	FBXO38
CYP2B6	MED13L	LSM5	DENR
DPP4	IDS	NBN	ANGEL2
HSD17B6	GNS	MTIF2	N4BP2L2
MBIP	ATP6V0A1	PSMA4	MCM4
HNRNPA2B1	SIDT2	TBCA	EZH1
DBT	DNAJC21	CLPX	KIAA0240
SNRPG			

Table S4: Data example–Positive control genes related to lung cancer.

KEGG Pathways	Bayesian	MAPE_I_Fisher	MAPE_I_minP
	$\hat{\phi}_g$	Q-value†	Q-value†
Natural Killer Cell Mediated Cytotoxicity	1	0*	0.011*
Nucleotide Excision Repair	1	0*	0.011*
Non-Small Cell Lung Cancer	1	0.015*	0.020*
DNA Replication	1	0.004*	0*
Thyroid Cancer	1	0*	0.906
Pathways In Cancer	1	0*	0.940
T Cell Receptor Signaling Pathway	1	0.004*	0.690
Melanoma	1	0.009*	0.641
PPAR Signaling Pathway	1	0.011*	1
Renal Cell Carcinoma	1	0.012*	0.970
mTOR Signaling Pathway	1	0.033*	0.926
Pancreatic Cancer	1	0.035*	0.949
Small Cell Lung Cancer	1	0.045*	0.822
VEGF Signaling Pathway	1	0.050	0.760
TGF Beta Signaling Pathway	1	0.316	0.513

†:Q-values with "*" are below the 0.05 threshold value.

Table S5: Selected enriched pathways identified by the Bayesian model.

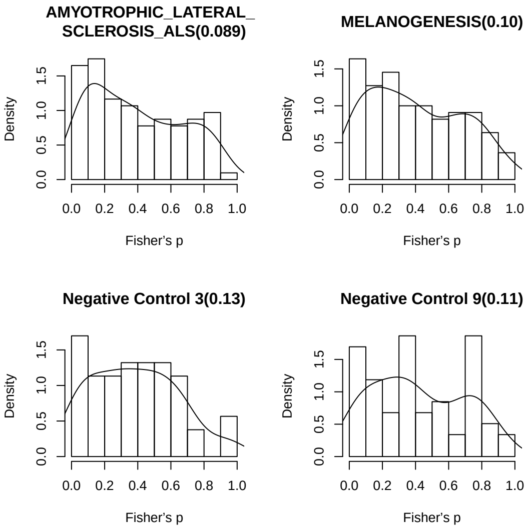

Figure S6: Data example–Empirical distributions of p-values from the Fisher's combined probability tests for genes in selected pathways with low estimated posterior probability of enrichment. For each pathway/gene set, the number in the parenthesis is the estimate of the corresponding posterior enrichment probability.

We also carried out the following analysis for the data example, as suggested by one of the reviewers. First, we randomly split each data set in two with the requirement that both groups will have at least three samples. Then we ran our proposed method and the MAPEs at their default settings. After obtaining an ordered list of pathways from each method, we calculated the percent of common pathways identified in the two halves among the top 5%, 10%, 15% and 20% pathways, respectively. We repeated this procedure 10 times and report the average percent values of pathways in common in the table below. The result shows that the reproducibility of the Bayesian method appears to be better or in par with the MAPE ones.

Top Pathways(%)	MAPE_P	MAPE_G	MAPE_I	Bayesian
5%	10.0%	3.0%	6.0%	31.7%
10%	23.5%	13.5%	11.5%	45.0%
15%	42.3%	17.0%	23.7%	45.0%
20%	49.8%	20.5%	28.8%	46.3%

Table S6: Data example–Comparison in percentage of common pathways identified based on random half splits.

References

Bild, A. H., Yao, G., Chang, J. T., Wang, Q., Potti, A., Chasse, D., Joshi, M.-B., Harpole, D., Lancaster, J. M., Berchuck, A., Olson, J. A., Jr, Marks, J. R., Dressman, H. K., West, M., and Nevins, J. R. (2006). Oncogenic pathway signatures in human cancers as a guide to targeted therapies. *Nature*, 439(19):353–357.

Kuner, R., Muley, T., Meister, M., Ruschhaupt, M., Buness, A., Xu, E., Schnabel, P., Warth, A., Poustka, A., Sültmann, H., and Hoffmann, H. (2009). Global gene expression analysis reveals specific patterns of cell junctions in non-small cell lung cancer subtypes. *Lung Cancer*, 63(1):32–38.

Potti, A., Mukherjee, S., Petersen, R., Dressman, H., Bild, A., Koontz, J., Kratzke, R., Watson, M., Kelley, M., Ginsburg, G., West, M., Harpole, D. J., and Nevins, J. (2006). A genomic strategy to refine prognosis in early-stage non-small-cell lung cancer. *N Engl J Med.*, 355(6):570–80.

Shedden, K., Taylor, J. M. G., Enkemann, S. A., Tsao, M.-S., Yeatman, T. J., Gerald, W. L., Eschrich, S., Jurisica, I., Giordano, T. J., Misek, D. E., Chang, A. C., Zhu, C. Q., Strumpf, D., Hanash, S., Shepherd, F. A., Ding, K., Seymour, L., Naoki, K., Pennell, N., Weir, B., Verhaak, R., Ladd-Acosta, C., Golub, T., Gruidl, M., Sharma, A., Szoke, J., Zakowski, M., Rusch, V., Kris, M., Viale, A., Motoi, N., Travis, W., Conley, B., Seshan, V. E., Meyerson, M., Kuick, R., Dobbin, K. K., Lively, T., Jacobson, J. W., and Beer, D. G. (2008). Gene expressionšcbased survival prediction in lung adenocarcinoma: a multi-site, blinded validation study. *NATURE MEDICINE*, 14(8):822–827.

Tibshirani, R., Hastie, T., Narasimhan, B., and Chu, G. (2002). Diagnosis of multiple cancer types by shrunken centroids of gene expression. *Proc Natl Acad Sci U S A*, 99(10):6567–6572.

Tibshirani, R., Hastie, T., Narasimhan, B., and Chu, G. (2003). Class prediction by nearest shrunken centroids, with applications to DNA microarrays. *Statistical Science*, 18:104–117.

Zhu, C.-Q., Ding, K., Strumpf, D., Weir, B. A., Meyerson, M., Pennell, N., Thomas, R. K., Naoki, K., Ladd-Acosta, C., Liu, N., Pintilie, M., Der, S., Seymour, L., Jurisica, I., Shepherd, F. A., , and Tsao, M.-S. (2010). Prognostic and predictive gene signature for adjuvant chemotherapy in resected nonšcsmall-cell lung cancer. *Journal of Clinical Oncology*, 28(29):4417–4424.
